# Predictors of user satisfaction with forest healing services differ by health status

**DOI:** 10.3389/fpubh.2026.1850081

**Published:** 2026-07-02

**Authors:** Minji Kang, Jeonghee Lee, Hyun Jin Lee

**Affiliations:** Forest Human Service Division, Future Forest Strategy Department, National Institute of Forest Science, Seoul, Republic of Korea

**Keywords:** health status, multimorbidity, nature-based intervention, non-medical health resource, service satisfaction, tailored intervention

## Abstract

**Objective:**

Forest healing is increasingly recognized as a public health resource; however, empirical evidence remains limited on how the predictors of service satisfaction vary across users with different health profiles. This study investigated health-status-specific predictors of satisfaction with forest healing services across Korea's national Healing Forest sites.

**Methods:**

A cross-sectional survey was conducted among participants from 20 Healing Forest sites. Respondents were categorized into no-disease, single-disease, and multimorbidity groups based on self-reported chronic conditions. Multiple regression analyses were performed to examine factors associated with overall satisfaction within each health status group.

**Results:**

Program structure, perceived usefulness of activities, and instructor expertise significantly predicted satisfaction across all groups, with relative contributions varying by health status: program structure was most influential for those without disease, activity usefulness for those with a single disease, and instructor expertise for participants with multimorbidity. The forest environment received uniformly high ratings across all groups, suggesting environmental comfort functions as a necessary but non-differentiating condition. Overall satisfaction and most service-specific domains did not differ significantly across health status groups; information sufficiency and equipment appropriateness were the only domains showing statistically significant group differences, though effect sizes were negligible, reflecting different informational needs and perceived burdens across groups.

**Conclusion:**

The predictors of overall satisfaction with forest healing services differ by health status, highlighting the importance of differentiated, health-status-sensitive service models tailored to users' functional capacities and diverse disease burdens.

## 1 Introduction

Interest in slow aging, an emerging health practice aimed at decelerating biological aging through improved dietary patterns and lifestyle modifications, has expanded rapidly in recent years (1). This trend reflects a broader societal shift toward proactive and self-directed health management, rather than a temporary wellness movement. Simultaneously, the prevalence of chronic conditions such as hypertension, diabetes mellitus, and hyperlipidemia continues to rise. National surveillance data indicate that the number of individuals with diabetes has increased at an average annual rate of 4.4% over the past 5 years, with even faster growth among adults in their twenties (7.4%) and adolescents (5.5%) (2). These patterns demonstrate that chronic diseases are no longer confined to older adults but have emerged as pervasive life-course health risks. Despite advances in medical technology, noncommunicable diseases remain the leading cause of morbidity and mortality worldwide, accounting for approximately 75% of global deaths and substantially reducing healthy life expectancy (3). Similar trends are observed in Korea, where chronic conditions account for 78.1% of deaths and significantly reduce healthy life expectancy (1). Consequently, public health strategies increasingly emphasize prevention-oriented, multidimensional approaches that integrate physical, psychological, and social health domains (4).

Nature-based interventions have gained attention as complementary, non-medical health resources within these prevention frameworks (5, 6). Among them, forest healing has been widely studied, with evidence demonstrating that forest microclimates, biodiversity, and phytoncides reduce stress responses, enhance emotional wellbeing, and promote physiological regulation (7, 8). Research involving both clinical and subclinical populations—including individuals with cancer (9, 10), metabolic syndrome (11), and cardiopulmonary conditions (12–14)—has consistently highlighted the capacity of forest environments to support immune and metabolic processes relevant to chronic disease management.

Parallel to these scientific developments, many countries have begun integrating nature-based interventions into public health systems, with accumulating clinical evidence across diverse national contexts and participant populations supporting this expansion (5, 6, 15). Among these, Korea has developed a national model of structured forest healing through its designated Healing Forests, with 54 sites jointly operated by the Korea Forest Service, Korea Forest Welfare Institute, and local governments since 2009 to deliver programs tailored to demographic and health-related characteristics (16, 17). These programs combine physical, sensory, and psychological activities under the supervision of certified Forest Healing Instructors and are implemented within a standardized national evaluation framework to ensure service quality (18–20). Existing studies of these services have predominantly examined user satisfaction in relation to environmental and operational attributes such as facility quality, landscape conditions, accessibility, and service convenience (21–24).

However, as forest healing services increasingly reach populations with diverse health profiles, empirical studies have seldom examined how individual health-related heterogeneity influences satisfaction and service evaluation. Service satisfaction is a multidimensional construct influenced by both service attributes and user characteristics. Functional health status, perceived physical burden, and activity capacity may meaningfully contribute to satisfaction by shaping how users experience and interpret service quality (25, 26). Notably, forest therapy participants are health-wise heterogeneous, with evidence indicating that a substantial proportion live with at least one chronic condition (27), suggesting that health status represents a meaningful source of variation in service evaluation.

Health status, in particular, affects perceptions of physical safety, accessibility, and the appropriateness of activity intensity—components embedded directly in the experiential process of forest healing. Individuals with chronic conditions often experience accumulated physical burden and prolonged treatment histories, increasing sensitivity to environmental comfort, mobility demands, and exertional requirements during participation (28, 29). Those with multimorbidity may experience even greater functional limitations and altered thresholds for physical and sensory stimuli, suggesting that their evaluative criteria for service quality differ substantially from those of healthier participants (30–32). Despite the theoretical and clinical significance of these differences, satisfaction analyses rarely account for individual-level health heterogeneity, leaving a critical empirical gap. Addressing this gap is essential for designing health-responsive, user-centered forest healing services and for accurately evaluating their public health potential.

Therefore, this study examined differences in satisfaction with forest healing services across three health status groups (no chronic disease, single chronic disease, and multimorbidity). We analyzed group-level differences in overall and domain-specific satisfaction and identified health status-specific predictors of satisfaction. The findings support the development of tailored forest healing services that accommodate diverse health-related needs and clarify the role of forest-based interventions as public health resources for chronic disease prevention and the extension of healthy life expectancy.

## 2 Materials and methods

### 2.1 Survey overview

This study utilized data from the 2024 Forest Healing Program User Satisfaction Survey conducted by the Korea Forest Service. The survey was administered between January and December 2024 across 20 national and public Healing Forest sites and targeted adults aged 19 years or older who participated in forest healing programs. Participants completed the questionnaire via an online link immediately following program completion. A total of 4,600 responses were collected; 375 were excluded due to incomplete or insincere responses, including responses with missing data on key items and patterned or inconsistent response sets, resulting in a final sample of 4,225. All data were fully de-identified secondary data provided by the Korea Forest Service prior to transfer to the research team, with no personally identifiable information included. Participants provided informed consent upon survey completion through the Korea Forest Service's official survey platform and were notified that their anonymized responses may be used for research purposes. As this study utilized de-identified administrative data collected by a public institution under its statutory mandate, it satisfied the criteria for exemption from institutional review board review under Article 2, Clause 1 of the Korean Bioethics and Safety Act.

### 2.2 Measurement instrument

The structured questionnaire comprised items on demographic characteristics (four items), participation characteristics (four items), overall and domain-specific service satisfaction (11 items), and one open-ended item soliciting suggestions for improvement (Table 1). Service-related domains included program structure, perceived usefulness of activities, instructor professionalism, information sufficiency, and reservation/access convenience. All closed-ended items were rated on a 7-point Likert scale (1 = very dissatisfied, 7 = very satisfied). The open-ended item allowed respondents to freely describe their needs or recommendations for enhancing forest healing services. participants were classified into three health status groups—no disease, single disease, and multimorbidity—based on self-reported chronic conditions. Disease names were standardized using the International Classification of Diseases (ICD-10) and the Korean Standard Classification of Diseases (KCD-7/8). Chronic diseases were categorized according to the World Health Organization and the Korea Community Health Survey, including hypertension, diabetes mellitus, stroke, chronic respiratory diseases, and mental health–related conditions (33, 34). Additional chronic conditions identified by the Korea Disease Control and Prevention Agency—such as dyslipidemia, arthritis, chronic kidney disease, and thyroid disorders—were classified as “Other chronic diseases” (34). Respondents reporting no chronic conditions were assigned to the no-disease group, those reporting a single condition to the single-disease group, and those reporting two or more chronic conditions to the multimorbidity group.

**Table 1:** Survey items and response format.

Variables	Items
1. Demographic characteristics	Gender, age, residential area, chronic disease (multiple responses)
2. Participation characteristics	Awareness source, participation purpose, companion type, key considerations
3. Service satisfaction	11 items (7-point Likert scale)
Overall satisfaction	Q1. I am satisfied with the overall program.
Session duration adequacy	Q2. The program duration was appropriate.
Program structure appropriateness	Q3. The program structure was appropriate.
Perceived usefulness of activities	Q4. The program content was beneficial.
Group size adequacy	Q5. The number of participants was appropriate.
Instructor expertise	Q6. The instructor had sufficient expertise.
Reservation and participation convenience	Q7. The reservation and participation process was convenient.
Information sufficiency	Q8. The pre-visit information was sufficient.
Equipment appropriateness	Q9. The materials and equipment were appropriate.
Environmental comfort and harmony	Q10. The site was comfortable and harmonized with the natural environment.
Amenities accessibility	Q11. The facilities were convenient to use.
4. Suggestions for improvement	Open-ended items regarding improvement suggestions for service satisfaction

### 2.3 Statistical analysis

All statistical analyses were performed using IBM SPSS Statistics (version 20.0; IBM Corp., Armonk, NY, USA). Construct validity of the satisfaction items was assessed using factor analysis, and internal consistency was evaluated with Cronbach's α. Descriptive statistics and frequency analyses were used to summarize participants' demographic and program participation characteristics. Differences in satisfaction across health status groups were examined using the Kruskal–Wallis test, followed by Dunn–Bonferroni *post hoc* comparisons where appropriate. Multiple linear regression analysis was conducted to identify factors associated with overall satisfaction. Overall satisfaction served as the dependent variable, while domain-specific satisfaction scores—including program structure, perceived usefulness of activities, instructor professionalism, information sufficiency, and reservation/access convenience—were included as independent variables. To formally compare standardized regression coefficients across health status groups, the Paternoster Z-test was applied for all three pairwise group comparisons (35). The standard errors of standardized coefficients were derived from the unstandardized standard errors using SE(β) = SE(B) × SD(X)/SD(Y). To assess the robustness of the regression findings, sensitivity analyses were conducted by re-estimating all models with gender and age as additional covariates. Mixed-effects models with site as a random effect were also fitted to account for potential clustering across the 20 Healing Forest sites. Statistical significance was set at *p* < 0.05, and results presented as mean ± standard deviation (mean ± SD).

### 2.4 Qualitative content analysis of open-ended responses

Open-ended responses were analyzed using qualitative content analysis. Invalid responses (e.g., blank entries, “none,” or non-meaningful text) were removed prior to analysis. Meaningful responses were segmented into meaning units based on shifts in semantic content, and these units were reviewed iteratively and coded using open coding to derive detailed categories. When a single response contained multiple suggestions for improvement, it was divided into separate meaning units and coded individually. To enhance coding reliability, 10%−20% of meaning units were randomly selected and independently coded by a second researcher. Inter-coder reliability was assessed using Cohen's κ, yielding κ =.68 at the detailed category level and κ =.82 at the broader thematic level, indicating substantial agreement. Discrepancies were resolved through discussion. Comparisons were conducted across the three health status groups (no disease, single disease, and multimorbidity). For supplementary analysis, the single-disease and multimorbidity groups were combined into a single chronic disease group, as the number of qualitative responses from the multimorbidity group alone (*n* = 33) was insufficient to support independent group analysis. Additionally, a basic keyword analysis was performed to explore differences in textual expressions, which involved removing non-Korean characters, tokenizing text, and comparing relative word frequencies across groups. All qualitative analyses were descriptive and did not involve statistical testing.

## 3 Results

### 3.1 Demographic characteristics and participation characteristics

Table 2 presents the demographic and participation characteristics of the 4,225 respondents. Females comprised 65.7% of the sample, exceeding males (34.3%). Individuals in their 50s represented the largest age group (29.0%), followed by those aged 60 years or older (24.8%) and those in their 40s (23.0%), indicating a predominance of middle-aged and older adults. The most common residential regions were Gyeonggi (31.7%), Seoul (21.3%), Jeonbuk (12.5%), and Chungnam (6.8%). Regarding health status, 58.1% of respondents were classified in the no-disease group, 32.6% in the single-disease group, and 9.3% in the multimorbidity group. The most frequently reported chronic conditions were hypertension (16.3%), obesity (9.2%), mood and stress-related disorders (6.8%), and diabetes mellitus (6.8%). Participation characteristics showed that most respondents first learned about forest healing services through recommendations from acquaintances (73.0%), followed by public institution websites (14.4%) and web portals (7.8%). The primary reasons for participation included emotional wellbeing (26.7%), physical health improvement (20.3%), social interaction (17.7%), and experiential activities (17.1%). Group participation accounted for the highest proportion (51.4%), followed by participation with family members (27.4%) and with friends or partners (19.6%). When choosing to participate, the most influential considerations were program content (46.6%) and environmental attributes such as comfort and natural setting (38.2%), whereas travel time (6.5%) and program cost (4.0%) were less influential.

**Table 2:** Demographic and participation characteristics (*n* = 4,225).

Variables		*N*	%
Gender	Male	1,448	34.3
	Female	2,777	65.7
Age	20s	358	8.5
	30s	619	14.7
	40s	973	23.0
	50s	1,226	29.0
	60s ≤	1,049	24.8
Residential area	Seoul	899	21.3
	Busan	38	0.9
	Incheon	109	2.6
	Daegu	197	4.7
	Daejeon	55	1.3
	Gwangju	124	2.9
	Ulsan	14	0.3
	Sejong	41	1.0
	Gyeonggi	1,341	31.7
	Gangwon	33	0.8
	Chungbuk	135	3.2
	Chungnam	289	6.8
	Jeonbuk	527	12.5
	Jeonnam	261	6.2
	Gyeongbuk	101	2.4
	Gyeongnam	33	0.8
	Jeju	28	0.7
Chronic disease status	No disease	2,455	58.1
	Single disease	1,378	32.6
	Multimorbidity	392	9.3
Major types of chronic diseases^*^	Hypertension	690	16.3
	Cerebrovascular disease	54	1.3
	Heart disease	51	1.2
	Diabetes mellitus	288	6.8
	Obesity	388	9.2
	Asthma	60	1.4
	COPD/chronic bronchitis	38	0.9
	Chronic sinusitis	83	2.0
	Dermatitis	124	2.9
	Mood- and stress-related disorder	288	6.8
	Malignant neoplasms	66	1.6
	Others	167	3.9
Awareness source	Acquaintances	3,085	73.0
	Mass media	107	2.5
	Web portals	330	7.8
	Social media	96	2.3
	Public institution websites	607	14.4
Participation purpose	Forest appreciation	502	11.9
	Experiential activities	722	17.1
	Eco facilities	106	2.5
	Social interaction	748	17.7
	Physical health improvement	856	20.3
	Emotional wellbeing	1,126	26.7
	Self-reflection	165	3.9
Companion type	Alone	70	1.6
	Family	1,156	27.4
	Friends/partner	827	19.6
	Group	2,172	51.4
Key considerations	Program cost	170	4.0
	Program content	1,969	46.6
	Travel time	275	6.5
	Transportation access	197	4.7
	Environment attributes	1,614	38.2

^*^Percentages are based on the total number of respondents. Multiple responses were allowed; therefore, the total percentage may have exceeded 100%.

### 3.2 Comparison of overall and service-specific satisfaction by health status

Principal component analysis and reliability testing were conducted to assess the construct validity and internal consistency of the satisfaction scale. All 11 items loaded onto a single factor (eigenvalue = 7.533), explaining 68.48% of the total variance. Factor loadings ranged from 0.76 to 0.89, exceeding the recommended threshold of 0.70. The Kaiser–Meyer–Olkin value indicated excellent sampling adequacy (0.957), and Bartlett's test of sphericity was statistically significant (χ^2^(55) = 40,238.48, *p* < 0.001). Reliability analysis confirmed high internal consistency (Cronbach's α = 0.94) (see Table 3). Across all items, mean scores for overall satisfaction and the 10 service-specific domains exceeded 6.4 on a 7-point scale, indicating generally high levels of satisfaction. The highest ratings were observed for instructor expertise (6.74), environmental comfort and harmony (6.74), and perceived usefulness of activities (6.70), with these patterns consistent across all health status groups. The Kruskal–Wallis test identified statistically significant differences for information sufficiency (χ^2^)(2) = 8.65, *p* < 0.05) and equipment appropriateness (χ^2^(2) = 15.10, *p* < 0.01). For session duration, program structure, group size adequacy, reservation convenience, amenity accessibility, and other domains, mean satisfaction tended to be higher among individuals with better health (i.e., fewer chronic conditions), although these differences were not statistically significant (see Table 4).

**Table 3:** Factor and reliability analysis of service satisfaction items.

Item description	Communality	Factor loading	If deleted	Cronbach's α
Q1. Overall satisfaction	0.678	0.892	0.947	0.951
Q2. Session duration adequacy	0.663	0.881	0.947	
Q3. Program structure appropriateness	0.795	0.868	0.944	
Q4. Perceived usefulness of activities	0.777	0.849	0.944	
Q5. Group size adequacy	0.672	0.824	0.947	
Q6. Instructor expertise	0.720	0.820	0.946	
Q7. Reservation and participation convenience	0.626	0.820	0.948	
Q8. Information sufficiency	0.578	0.814	0.950	
Q9. Equipment appropriateness	0.754	0.791	0.945	
Q10. Environmental comfort and harmony	0.672	0.773	0.947	
Q11. Amenities accessibility	0.597	0.760	0.949	

Extraction method: principal component analysis. One factor extracted. Kaiser–Meyer–Olkin = 0.957; Bartlett's Test of Sphericity: χ^2^ (55) = 40238.48, *p* < 0.001.

Eigenvalue = 7.533; Variance explained = 68.48%.

**Table 4:** Service satisfaction scores and group comparisons by health status.

Item Description	Total	No disease	Single disease	Multi- morbidity	χ^2^(2)	*p*	η^2^
Q1. Overall satisfaction	6.728 ± 0.606	6.742 ± 0.587	6.711 ± 0.602	6.696 ± 0.724	4.317	.116	.001
Q2. Session duration adequacy	6.632 ± 0.703	6.650 ± 0.675	6.615 ± 0.726	6.582 ± 0.782	2.660	.264	.000
Q3. Program structure appropriateness	6.648 ± 0.678	6.667 ± 0.653	6.625 ± 0.695	6.610 ± 0.766	4.075	.130	.001
Q4. Perceived usefulness of activities	6.697 ± 0.630	6.711 ± 0.620	6.680 ± 0.626	6.671 ± 0.698	4.394	.111	.001
Q5. Group size adequacy	6.650 ± 0.671	6.663 ± 0.649	6.636 ± 0.691	6.617 ± 0.737	1.732	.421	.000
Q6. Instructor expertise	6.736 ± 0.592	6.748 ± 0.574	6.723 ± 0.605	6.709 ± 0.653	2.741	.254	.000
Q7. Reservation and participation convenience	6.607 ± 0.733	6.625 ± 0.706	6.589 ± 0.760	6.554 ±0.801	3.070	.215	.000
Q8. Information sufficiency	6.545 ± 0.827	6.576 ± 0.794^a^	6.524 ± 0.843^ab^	6.431 ± 0.949^b^	8.651	.013	.002
Q9. Equipment appropriateness	6.656 ± 0.667	6.685 ± 0.629^a^	6.630 ± 0.707^ab^	6.561 ± 0.741^b^	15.104	.001	.003
Q10. Environmental comfort and harmony	6.743 ± 0.560	6.751 ± 0.548	6.739 ± 0.552	6.707 ± 0.650	1.297	.523	.000
Q11. Amenities accessibility	6.624 ± 0.718	6.638 ± 0.690	6.611 ± 0.741	6.582 ± 0.802	.921	.631	.000

Values are presented as mean ± standard deviation. Differences among health status groups were examined using the Kruskal–Wallis test. Post hoc pairwise comparisons were conducted using Dunn's test with Bonferroni correction. η^2^ = eta-squared effect size; values < 0.01 indicate negligible effect. Different superscripts (a, b) indicate statistically significant differences between groups.

### 3.3 Factors affecting overall satisfaction by health status

Multiple linear regression analyses were conducted to examine factors associated with overall satisfaction within each health status group. All models were statistically significant (no disease: *F* = 629.78, *p* < 0.001; single disease: *F* = 235.40, *p* < 0.001; multimorbidity: *F* = 55.95, *p* < 0.001). Durbin–Watson statistics ranged from 1.836 to 2.013, indicating that the assumption of independent errors was satisfied (see Tables 5–7). Assumptions of linearity, homoscedasticity, and normality of residuals were also met, supporting the reliability of the analytical results. Across all groups, program structure and perceived usefulness of activities consistently exhibited the highest regression coefficients, confirming their roles as the strongest predictors of overall satisfaction. The following subsections present detailed regression findings for each health status group.

**Table 5:** Multiple linear regression analysis of factors associated with overall satisfaction — no disease group (*N* = 2,455).

Predictor^a^	*B*	SE B	β	95% CI^b^	*t*	*p*
(Constant)^c^	0.838	0.083	–	–	10.064	0.000
Q2. Session duration adequacy	0.133	0.015	.153	[0.104, 0.162]	8.678	0.000
Q3. Program structure appropriateness	0.311	0.021	.346	[0.270, 0.352]	14.991	0.000
Q4. Perceived usefulness of activities	0.233	0.020	.247	[0.194, 0.272]	11.410	0.000
Q5. Group size adequacy	−0.027	0.016	−0.029	[−0.058, 0.004]	−1.648	0.100
Q6. Instructor expertise	0.147	0.021	0.143	[0.106, 0.188]	7.087	0.000
Q7. Reservation and participation convenience	−0.001	0.015	−0.001	[−0.030, 0.028]	−0.047	0.962
Q8. Information sufficiency	0.000	0.013	0.000	[−0.025, 0.025]	0.023	0.981
Q9. Equipment appropriateness	0.010	0.020	0.011	[−0.029, 0.049]	0.497	0.619
Q10. Environmental comfort and harmony	0.106	0.020	0.099	[0.067, 0.145]	5.220	0.000
Q11. Amenities accessibility	−0.033	0.014	−0.038	[−0.060, −0.006]	−2.298	0.022
*F*(*p*) = 629.777^***^, adj. *R*^2^ = 0.719, Durbin-Watson = 2.013						

^a^Service satisfaction domains (Q2–Q11) served as independent variables. ^***^p < 0.001. ^b^95% CI = 95% confidence interval for unstandardized coefficient B. ^c^Dependent variable: Q1. Overall satisfaction.

**Table 6:** Multiple linear regression analysis of factors associated with overall satisfaction — Single disease group (*N* = 1,378).

Predictor^a^	*B*	SE B	β	95% CI^b^	*t*	*p*
(Constant)^c^	1.121	0.131	–	–	8.553	0.000
Q2. Session duration adequacy	0.145	0.024	0.176	[0.100, 0.194]	6.118	0.000
Q3. Program structure appropriateness	0.153	0.030	0.176	[0.094, 0.212]	5.027	0.000
Q4. Perceived usefulness of activities	0.314	0.032	0.327	[0.249, 0.375]	9.724	0.000
Q5. Group size adequacy	−0.014	0.023	−0.016	[−0.060, 0.030]	−0.615	0.539
Q6. Instructor expertise	0.132	0.026	0.132	[0.081, 0.183]	5.037	0.000
Q7. Reservation and participation convenience	0.031	0.020	0.038	[−0.008, 0.070]	1.578	0.115
Q8. Information sufficiency	0.007	0.018	0.009	[−0.028, 0.042]	0.368	0.713
Q9. Equipment appropriateness	−0.052	0.024	−0.062	[−0.099, −0.005]	−2.153	0.031
Q10. Environmental comfort and harmony	0.102	0.030	0.093	[0.043, 0.161]	3.374	0.001
Q11. Amenities accessibility	0.020	0.021	0.025	[−0.019, 0.063]	0.984	0.325
*F*(*p*) = 235.402^***^, adj. *R*^2^ =.630, Durbin-Watson = 1.956						

^a^Service satisfaction domains (Q2–Q11) served as independent variables. ^***^p < 0.001. ^b^95% CI = 95% confidence interval for unstandardized coefficient B. ^c^Dependent variable: Q1. Overall satisfaction.

**Table 7:** Multiple linear regression analysis of factors associated with overall satisfaction — multimorbidity group (*N* = 392).

Predictor^a^	*B*	SE B	β	95% CI^b^	*t*	*p*
(Constant)^c^	0.875	0.279	–	–	3.134	0.002
Q2. Session duration adequacy	0.066	0.044	0.071	[−0.023, 0.153]	1.475	0.141
Q3. Program structure appropriateness	0.236	0.070	0.250	[0.101, 0.377]	3.368	0.001
Q4. Perceived usefulness of activities	0.185	0.073	0.179	[0.047, 0.335]	2.537	0.012
Q5. Group size adequacy	0.010	0.051	0.011	[−0.091, 0.109]	0.203	0.839
Q6. Instructor expertise	0.340	0.059	0.307	[0.218, 0.454]	5.757	0.000
Q7. Reservation and participation convenience	0.020	0.046	0.022	[−0.063, 0.117]	0.426	0.671
Q8. Information sufficiency	−0.089	0.038	−0.117	[−0.168, −0.018]	−2.355	0.019
Q9. Equipment appropriateness	0.105	0.061	0.108	[−0.016, 0.228]	1.714	0.087
Q10. Environmental comfort and harmony	−0.006	0.059	−0.002	[−0.121, 0.115]	−0.098	0.922
Q11. Amenities accessibility	0.005	0.047	0.006	[−0.092, 0.092]	0.107	0.915
*F*(*p*) = 55.946^***^, adj. *R*^2^ =.584, Durbin-Watson = 1.836						

^a^Service satisfaction domains (Q2–Q11) served as independent variables. ^***^p < 0.001. ^b^95% CI = 95% confidence interval for unstandardized coefficient B. ^c^Dependent variable: Q1. Overall satisfaction.

#### 3.3.1 No-disease group

Among individuals without chronic disease, significant predictors of overall satisfaction included program structure (β = 0.346, *p* < 0.001), perceived usefulness of activities (β = 0.247, *p* < 0.001), session duration adequacy (β = 0.153, *p* < 0.001), instructor expertise (β = 0.143, *p* < 0.001), and environmental comfort and harmony (β = 0.099, *p* < 0.001). The model demonstrated high explanatory power (adjusted *R*^2^ = 0.719), and variance inflation factor values ranged from 2.17 to 5.18, indicating no multicollinearity concerns.

#### 3.3.2 Single-disease group

For participants in the single-disease group, significant positive predictors of overall satisfaction included perceived usefulness of activities (β = 0.327, *p* < 0.001), program structure (β = 0.176, *p* < 0.001), session duration adequacy (β = 0.176, *p* < 0.001), instructor expertise (β = 0.132, *p* < 0.001), and environmental comfort and harmony (β = 0.093, *p* < 0.01). Equipment appropriateness demonstrated a small but significant negative association (β = −0.062, *p* < 0.05). The model explained a substantial proportion of variance (adjusted *R*^2^ = 0.630).

#### 3.3.3 Multimorbidity group

In the multimorbidity group, significant positive predictors of overall satisfaction included instructor expertise (β = 0.307, *p* < 0.001), program structure (β = 0.250, *p* < 0.01), and perceived usefulness of activities (β = 0.179, *p* < 0.05). In contrast, information sufficiency exhibited a significant negative association (β = −0.117, *p* < 0.05). The model demonstrated acceptable explanatory power (adjusted *R*^2^ = 0.584).

#### 3.3.4 Sensitivity analyses

To assess the robustness of the regression findings, two sensitivity analyses were conducted. First, ordinal logistic regression models were estimated for each health status group to account for the bounded 7-point Likert scale and strong ceiling effects. The direction and significance of the main predictors were broadly consistent across OLS and ordinal models in all three groups: program structure, perceived usefulness of activities, and instructor expertise remained the strongest predictors in the no-disease, single-disease, and multimorbidity groups, respectively (Supplementary Table 1), supporting the robustness of the linear regression approach. Second, all models were re-estimated with gender and age included as additional covariates. Standardized regression coefficients for the key predictors were virtually unchanged across all groups (maximum Δβ = 0.012), indicating that the original findings were not attributable to demographic confounding. Mixed-effects models incorporating site as a random effect yielded negligible random-effect variance (close to zero) across all groups, indicating that site-level clustering did not substantively influence the results (Supplementary Table 2).

#### 3.3.5 Between-group comparisons of regression coefficients

Pairwise comparisons of standardized regression coefficients using the Paternoster *Z*-test revealed statistically significant between-group differences for several service domains (Supplementary Table 3). Program structure was significantly more influential in the no-disease group than in the single-disease group (*Z* = 4.058, *p* < 0.001), while perceived usefulness of activities was significantly more influential in the single-disease group than in the no-disease group (*Z* = −2.003, *p* = 0.045). Instructor expertise demonstrated a consistent gradient across health status groups, with significantly stronger associations in the multimorbidity group compared with both the no-disease group (*Z* = −2.868, *p* = 0.004) and the single-disease group (*Z* = −2.940, *p* = 0.003). Information sufficiency was negatively associated with overall satisfaction in the multimorbidity group, and this negative association was significantly stronger than in both the no-disease group (*Z* = 2.229, *p* = 0.026) and the single-disease group (*Z* = 2.260, *p* = 0.024).

### 3.4 Qualitative content analysis of improvement requests by health status

Of the 1,240 participants who provided open-ended responses, 908 expressed overall satisfaction and were excluded from further analysis. The remaining 332 responses containing specific improvement requests were segmented into meaning units and analyzed, yielding eight axial categories (see Table 8). Representative translated quotes for each category are provided in Supplementary Table 4. Overall, the most frequently identified category was program content diversity and depth (87 cases; 26.2%), followed by program time and operational adequacy (56 cases; 16.9%), basic convenience infrastructure (52 cases; 15.7%), promotion and service outreach (42 cases; 12.6%), and spatial safety and environmental improvement (35 cases; 10.5%). Additional categories included accessibility and mobility convenience (33 cases; 9.9%), information, guidance, and reservation accessibility (17 cases; 5.1%), and instructor competence and operational expertise (10 cases; 3.0%). When comparing health status groups, the no-disease group submitted 168 improvement requests, whereas the combined chronic disease group (single and multiple chronic conditions) submitted 164. In both groups, program content diversity and depth accounted for the highest proportion, representing 24.4% of the no-disease group (41 cases) and 28.0% of the chronic disease group (46 cases). Within this category, the no-disease group more frequently requested experiential content (16 cases), whereas the chronic disease group emphasized rest and recovery-oriented content (12 cases).

**Table 8:** Open and axial coding results of improvement requests by health status group.

Axial coding	Open coding	No disease (*n*)	Chronic disease (*n*)
A. Program time and operational adequacy	Residential forest healing programs	2	7
	Participant group size adjustment	1	1
	Diversity of program time structure	2	1
	Program duration extension	22	13
	Program operation frequency	1	4
	Program pace control	1	1
B. Accessibility and mobility convenience	Transportation accessibility	2	2
	Barrier-free access	0	3
	Walking distance burden	4	3
	Parking convenience	4	4
	Wayfinding and signage systems	2	9
C. Basic convenience Infrastructure	Drinking water supply facilities	3	7
	Food and beverage infrastructure	1	6
	Sanitation facilities	1	2
	Hygiene supplies provision	2	0
	Overall facility conditions	4	2
	Pest control and environmental comfort facilities	4	1
	Restroom convenience	3	5
	Resting space availability	7	4
D. Program content diversity and depth	Age-specific program design	3	3
	Program content diversity	8	13
	Program content quality	2	4
	Diversity of program activity locations	5	6
	Experiential content	16	8
	Rest and recovery-oriented content	7	12
	Spatial and route safety management	4	9
E. Spatial safety and environmental improvement	Environmental maintenance of program spaces	12	10
	Pre-program and detailed information provision	7	1
F. Information, guidance, and reservation accessibility	Reservation system accessibility	3	5
	Reservation information accuracy	1	0
G. Promotion and service outreach	Promotion and information dissemination	27	15
H. Instructor competence and operational expertise	Instructor competence and operational expertise	7	3
Total		168	164

Group differences indicated descriptive comparisons only, and no statistical tests were performed.

In the accessibility and mobility convenience category, both groups reported comparable frequencies for transportation accessibility, parking convenience, and walking distance burden. Notably, requests for barrier-free access were observed exclusively in the chronic disease group (three cases). Program time and operational adequacy were also prominent, accounting for 29 cases (17.3%) in the no-disease group and 27 cases (16.5%) in the chronic disease group, with program duration extension being the most common specific request in both groups.

For basic convenience infrastructure, similar frequencies were observed across groups (25 cases in the no-disease group and 27 in the chronic disease group), with frequent references to drinking water supply, food and beverage facilities, restroom facilities, and resting spaces. Spatial safety and environmental improvement were more frequently reported in the chronic disease group (19 cases; 11.6%) than in the no-disease group (16 cases; 9.5%), particularly regarding spatial and route safety management and environmental maintenance of program spaces. Conversely, promotion and service outreach were substantially more prevalent in the no-disease group (27 cases; 16.1%) than in the chronic disease group (15 cases; 9.1%). Requests for information, guidance, and reservation accessibility were less frequent overall but more common in the no-disease group (11 cases) than in the chronic disease group (six cases), particularly regarding pre-program and detailed information. Finally, instructor competence and operational expertise were the least frequently reported categories in both groups, with seven cases in the no-disease group and three cases in the chronic disease group.

## 4 Discussion

This study examined the structure and predictors of service satisfaction across health status groups in the context of forest healing services provided through Korea's national Healing Forest program. Across all groups, satisfaction with environmental comfort, instructor expertise, and content usefulness was consistently high, and overall satisfaction did not differ significantly across health status groups. Information sufficiency and equipment appropriateness were the only service-specific domains showing statistically significant group differences, though effect sizes were negligible (information sufficiency: χ^2^(2) = 8.65, *p* < 0.05, η^2^ = 0.002; equipment appropriateness: χ^2^(2) = 15.10, *p* < 0.01, η^2^ = 0.003). Nevertheless, the predictors of overall satisfaction showed health-status-specific patterns, with program structure, content usefulness, and instructor expertise differing in their relative importance across groups.

Under the Act on the Promotion of Forest Culture and Recreation, Healing Forests are legally defined as spaces dedicated to forest healing activities, and Forest Healing Instructors are certified professionals responsible for program operation and participant guidance (16). This institutional framework demonstrates that forest healing services comprise integrated environmental resources, structured program content, and professional facilitation rather than mere exposure to natural settings. This conceptualization aligns with prior research indicating that forest environmental characteristics and programmatic structure shape users' experiences (18), activity-based program design influences perceived service quality (36), and instructors' professional judgment and relational skills mediate therapeutic outcomes (37). The consistently high ratings for environmental comfort, content usefulness, and instructor expertise observed in this study reinforce their roles as core components of Healing Forest service quality.

Although mean satisfaction scores tended to be higher in the no-disease group and lower in the multimorbidity group, these differences were largely non-significant. Prior studies suggest that an increasing number of chronic conditions is associated with lower subjective evaluations (30, 31), possibly due to accumulated functional limitations or perceived daily burden influencing cognitive appraisal (32, 38). Nevertheless, overall satisfaction remained uniformly high across all groups, suggesting that Healing Forest services were consistently delivered under established operational guidelines in a stable and standardized manner.

Healing Forest operations follow institutional standards regarding facility safety, program design, operational procedures, and qualified staffing, ensuring that core elements consistently meet minimum quality thresholds (39). Prior research on service quality indicates that physical environment, service delivery procedures, and staff expertise function as basic quality factors that, when adequately met, reduce the likelihood of between-group variations (40). When these foundational factors consistently exceed minimum thresholds, their discriminative power across user groups diminishes (41–43). Therefore, the limited group differences observed likely reflect a well-regulated operational system in which foundational quality is relatively stable and consistently upheld.

In contrast, regression analyses revealed meaningful differences in how service factors predicted overall satisfaction. Program structure and content usefulness were significant predictors across all groups, although their relative influence varied: program structure was most influential in the no-disease group (β = 0.346), content usefulness in the single-disease group (β = 0.327), and instructor expertise in the multimorbidity group (β = 0.307). These findings indicate that the benefits of forest healing services depend not just on natural exposure but on how experiences are structured within programmatic frameworks (22, 23). This aligns with evidence demonstrating that the effectiveness of nature-based interventions varies according to program content, structure, and engagement modality (44–46), as well as Attention Restoration Theory, which posits that restorative effects are more reliable when paired with intentional activity design (47). Accordingly, forest healing services appear to operate through structured experiential frameworks rather than passive natural exposure, consistent with findings that structured interventions elicit psychological and physiological benefits more reliably (44, 48, 49).

The differentiated pattern across groups—emphasizing program structure in the no-disease group, content usefulness in the single-disease group, and instructor expertise in the multimorbidity group—suggests that health status shapes the evaluative framework through which services are interpreted. As functional limitations increase, users may shift from prioritizing structural coherence to valuing relational and emotional aspects. This indicates a move beyond uniform activity-based models toward layered intervention designs that engage cognitive, emotional, and physiological pathways differently depending on user characteristics.

Session duration significantly predicted satisfaction in the no-disease and single-disease groups (β = 0.153 and β = 0.176, respectively), but not in the multimorbidity group (β = 0.071, n.s.), suggesting that evaluations of time structuring vary by health status. Users with fewer limitations may value activity flow and efficiency, whereas individuals with multimorbidity may prioritize relational qualities—emotional reassurance, reliability, and interaction quality—over temporal efficiency. Consistent with this, instructor expertise had the strongest effect in the multimorbidity group, aligning with principles of patient-centered care, in which emotional stability and relational support have heightened importance among users with greater health burden (50–52). Between-group comparisons using the Paternoster *Z*-test confirmed that the association between instructor expertise and overall satisfaction was statistically stronger in the multimorbidity group than in both the no-disease and single-disease groups. The value of professional facilitation in this context is further corroborated by recent evidence that therapist-guided forest immersion produced greater improvements in state anxiety, self-esteem, and total mood disturbance compared to self-guided immersion, with guided sessions particularly reducing interindividual variability in outcomes (53) — suggesting that structured instructor involvement may be especially important for users whose health status renders their responses to forest environments more variable and less predictable.

Beyond instructor expertise, information sufficiency showed a notably different pattern across health status groups. The multimorbidity group reported the lowest satisfaction with information sufficiency, and unlike other service domains, information sufficiency was negatively associated with overall satisfaction exclusively in this group (β = −0.117, *p* < 0.05), with between-group comparisons confirming this negative association was significantly stronger than in both the no-disease and single-disease groups. Requests for more detailed pre-program guidance suggest that individuals with multimorbidity require more specific, comprehensible, and anxiety-reducing information. Prior research has shown that vague or overly general information increases cognitive load and lowers satisfaction (42, 54–56). Consequently, the negative association observed in this study likely reflects a functional mismatch between the information provided and the needs of users with greater health burden. Given the cross-sectional design, this mechanism could not be directly confirmed; future research should explore health-status-tailored information provision strategies for users with multimorbidity.

The qualitative patterns observed in users' improvement requests further contextualize the regression findings by illustrating how health status shapes evaluative priorities in forest healing services. Users without chronic disease more frequently emphasized experiential engagement and activity-oriented program content, whereas users with chronic disease expressed stronger preferences for rest- and recovery-centered experiences; these findings are consistent with the differentiated contribution of service factors across health groups. This pattern aligns with the greater importance of program structure and content usefulness observed in healthier groups and the heightened role of instructor expertise among users with multimorbidity. Additionally, concerns related to accessibility, route safety, and environmental maintenance were more salient among users with chronic disease, suggesting that as functional limitations accumulate, physical predictability and perceived safety become increasingly important components of service evaluation. Collectively, these qualitative patterns reinforce the interpretation that health status shapes not only the relative importance of service factors but also the evaluative criteria applied to them, providing a contextual bridge between the regression results and the need for differentiated, health-responsive service strategies.

Overall, these findings indicate that satisfaction with forest healing services is a multilayered evaluative process influenced by users' health status. In the context of a rising chronic disease burden, forest healing services should evolve toward nature-based interventions systematically tailored to individual health profiles. Future operational strategies should include: (1) structured time, intensity, and content design for users without chronic disease or with a single condition; (2) relationship-centered programs emphasizing instructor competencies for individuals with multimorbidity; and (3) approaches that minimize informational, physical, and environmental burdens for users with greater health burden. As program standardization progresses and instructors' competencies in disease understanding, emotional support, and risk management expand, Healing Forests hold substantial potential as preventive and restorative public health resources that complement existing medical systems. The health-status-specific satisfaction structures identified in this study can inform quality assurance systems, tailored service models, and integrated forest–health linkages.

## 5 Conclusions

This study found that the predictors of overall satisfaction with forest healing services differ by users' health status. While program structure, content usefulness, and instructor expertise were important predictors across all groups, their relative contributions differed among participants with no disease, a single disease, and multimorbidity. Environmental comfort received uniformly high ratings but did not significantly predict overall satisfaction, suggesting that the forest environment functions as a necessary precondition for service evaluation rather than as a direct contributor to satisfaction. Instead, higher-order service elements, including program design, activity structure, and the quality of instructor-participant interactions, are central to users' evaluations. These findings underscore the importance of adopting health-status-sensitive operational strategies for forest healing services. Practical implications include optimizing session time, intensity, and content for healthier participants; strengthening relationship-centered program delivery for individuals with multimorbidity; and reducing cognitive and physical burdens for users with greater health burden. Institutional efforts to standardize program quality and systematically enhance instructors' competencies in disease awareness, emotional support, and risk management may further strengthen the position of forest healing as a complementary public health resource.

This study has limitations due to its cross-sectional design and reliance on self-reported data, and the sample was restricted to adults aged 19 years or older, which limits generalizability to adolescent or younger populations. Disease severity, functional limitation, and treatment status were not assessed, and findings should be interpreted with this in mind. The primary analyses did not control for participation-related characteristics such as participation purpose, companion type, and program type, which should be considered when interpreting the findings. Future research should incorporate longitudinal methods, objective physiological and psychological measures including environmental exposure parameters, more refined analyses based on disease type and severity, and broader covariate adjustment to strengthen the interpretation of health-status-specific satisfaction patterns. Despite these limitations, the findings offer preliminary empirical evidence supporting health-status-based service differentiation and contribute to the development of evidence-informed forest healing services.

## Data Availability

The original contributions presented in the study are included in the article/Supplementary material, further inquiries can be directed to the corresponding authors.
