# Supplementary material for: Predictors of user satisfaction with forest healing services differ by health status
Source: Front Public Health. 2026 Jul 2;14:1850081. doi: 10.3389/fpubh.2026.1850081 (PMC13373039; doi:10.3389/fpubh.2026.1850081)
Supplement: Supplementary file 1 [file Table_1.docx]

**Supplementary Table 1.** Sensitivity analysis: comparison of OLS and ordinal logistic regression results by health status group.

| **Service domain** | | **OLS β** | **Ordinal B** | **SE** | ***p*** |
| --- | --- | --- | --- | --- | --- |
| ***No disease (n = 2,455)*** | | | | | |
|  | Q2. Session duration adequacy | 0.153*** | 0.852*** | 0.136 | 0.000 |
|  | Q3. Program structure appropriateness | 0.346*** | 1.660*** | 0.180 | 0.000 |
|  | Q4. Perceived usefulness of activities | 0.247*** | 1.150*** | 0.179 | 0.000 |
|  | Q5. Group size adequacy | −0.029 | −0.020 | 0.147 | 0.892 |
|  | Q6. Instructor expertise | 0.143*** | 0.479** | 0.168 | 0.004 |
|  | Q7. Reservation and participation convenience | −0.001 | 0.133 | 0.144 | 0.356 |
|  | Q8. Information sufficiency | 0.000 | 0.079 | 0.121 | 0.513 |
|  | Q9. Equipment appropriateness | 0.011 | 0.046 | 0.176 | 0.796 |
|  | Q10. Environmental comfort and harmony | 0.099*** | 0.622*** | 0.177 | 0.000 |
|  | Q11. Amenities accessibility | −0.038* | −0.216 | 0.149 | 0.147 |
| ***Single disease (n = 1,378)*** | | | | | |
|  | Q2. Session duration adequacy | .176*** | .879*** | .164 | .000 |
|  | Q3. Program structure appropriateness | .176*** | .805*** | .205 | .000 |
|  | Q4. Perceived usefulness of activities | .327*** | 1.444*** | .220 | .000 |
|  | Q5. Group size adequacy | -.016 | -.032 | .162 | .844 |
|  | Q6. Instructor expertise | .132*** | .655*** | .177 | .000 |
|  | Q7. Reservation and participation convenience | .039 | .153 | .152 | .314 |
|  | Q8. Information sufficiency | .009 | .164 | .131 | .210 |
|  | Q9. Equipment appropriateness | -.062* | -.199 | .166 | .229 |
|  | Q10. Environmental comfort and harmony | .093*** | .438* | .202 | .030 |
|  | Q11. Amenities accessibility | .025 | .034 | .160 | .834 |
| ***Multimorbidity (n = 392)*** | | | | | |
|  | Q2. Session duration adequacy | .071 | .493* | .225 | .029 |
|  | Q3. Program structure appropriateness | .250*** | .849* | .391 | .030 |
|  | Q4. Perceived usefulness of activities | .179* | .687 | .370 | .063 |
|  | Q5. Group size adequacy | .011 | .147 | .281 | .601 |
|  | Q6. Instructor expertise | .307*** | 1.380*** | .313 | .000 |
|  | Q7. Reservation and participation convenience | .022 | .078 | .275 | .775 |
|  | Q8. Information sufficiency | -.117* | -.504* | .247 | .042 |
|  | Q9. Equipment appropriateness | .108 | .961** | .344 | .005 |
|  | Q10. Environmental comfort and harmony | -.005 | -.243 | .326 | .455 |
|  | Q11. Amenities accessibility | .006 | -.014 | .253 | .954 |

OLS β = standardized OLS regression coefficient. Ordinal B = unstandardized ordinal logistic regression coefficient (logit link). SE = standard error. Directional inconsistencies were observed for two non-significant predictors only (Q7, no-disease group; Q11, multimorbidity group). * p < .05; ** p < .01; *** p < .001.
