# Supplementary material for: Predictors of user satisfaction with forest healing services differ by health status
Source: Front Public Health. 2026 Jul 2;14:1850081. doi: 10.3389/fpubh.2026.1850081 (PMC13373039; doi:10.3389/fpubh.2026.1850081)
Supplement: Supplementary file 2 [file Table_2.docx]

**Supplementary Table 2.** Sensitivity analysis: comparison of original and covariate-adjusted standardized regression coefficients (β) by health status group.

***Part a. Comparison of original and covariate-adjusted standardized regression coefficients (β) by health status group.***

| **Service domain** | | **Original β** | **Covariate-adjusted β** | **Δβ** |
| --- | --- | --- | --- | --- |
| ***No disease (n = 2,455)*** | | | | |
|  | Q2. Session duration adequacy | .153*** | .153*** | -.000 |
|  | Q3. Program structure appropriateness | .346*** | .348*** | .002 |
|  | Q4. Perceived usefulness of activities | .247*** | .243*** | -.004 |
|  | Q5. Group size adequacy | -.029 | -.030 | -.001 |
|  | Q6. Instructor expertise | .143*** | .140*** | -.003 |
|  | Q7. Reservation and participation convenience | -.001 | -.002 | -.001 |
|  | Q8. Information sufficiency | .000 | .001 | .001 |
|  | Q9. Equipment appropriateness | .011 | .018 | .007 |
|  | Q10. Environmental comfort and harmony | .099*** | .099*** | -.001 |
|  | Q11. Amenities accessibility | -.038* | -.037* | .002 |
|  | *Gender (ref: male)* | — | .004 | — |
|  | *Age* | — | .061*** | — |
| ***Single disease (n = 1,378)*** | | | | |
|  | Q2. Session duration adequacy | .176*** | .177*** | .002 |
|  | Q3. Program structure appropriateness | .176*** | .176*** | .000 |
|  | Q4. Perceived usefulness of activities | .327*** | .324*** | -.003 |
|  | Q5. Group size adequacy | -.016 | -.017 | -.001 |
|  | Q6. Instructor expertise | .132*** | .133*** | .000 |
|  | Q7. Reservation and participation convenience | .039 | .039 | -.000 |
|  | Q8. Information sufficiency | .009 | .010 | .001 |
|  | Q9. Equipment appropriateness | -.062* | -.061* | .000 |
|  | Q10. Environmental comfort and harmony | .093*** | .094*** | .000 |
|  | Q11. Amenities accessibility | .025 | .027 | .002 |
|  | *Gender (ref: male)* | — | .017 | — |
|  | *Age* | — | .013 | — |
| ***Multimorbidity (n = 392)*** | | | | |
|  | Q2. Session duration adequacy | .071 | .064 | -.006 |
|  | Q3. Program structure appropriateness | .250*** | .253*** | .003 |
|  | Q4. Perceived usefulness of activities | .179* | .184** | .005 |
|  | Q5. Group size adequacy | .011 | .014 | .004 |
|  | Q6. Instructor expertise | .307*** | .307*** | -.000 |
|  | Q7. Reservation and participation convenience | .022 | .023 | .001 |
|  | Q8. Information sufficiency | -.117* | -.115* | .002 |
|  | Q9. Equipment appropriateness | .108 | .104 | -.004 |
|  | Q10. Environmental comfort and harmony | -.005 | -.017 | -.012 |
|  | Q11. Amenities accessibility | .006 | .014 | .008 |
|  | *Gender (ref: male)* | — | .037 | — |
|  | *Age* | — | -.017 | — |

Original β = standardized OLS coefficient (service domains only). Covariate-adjusted β = standardized coefficient with gender and age added. Δβ = difference; maximum |Δβ| = 0.012. Gender: 0 = male, 1 = female. Age: ordinal scale (1 = 20s to 6 = 60s or older). — = not included in original model. * p < .05; ** p < .01; *** p < .001.

***Part b. Site-level random-effect variance from mixed-effects models by health status group.***

| **Health status group** | **Site random-effect variance** |
| --- | --- |
| No disease (n = 2,455) | Negligible (< 0.001) |
| Single disease (n = 1,378) | Negligible (< 0.001) |
| Multimorbidity (n = 392) | Negligible (< 0.001) |

Mixed-effects models with site as a random effect (REML). Negligible variance across all groups indicates site-level clustering did not substantively influence the results.
