# Supplementary material for: Predictors of user satisfaction with forest healing services differ by health status
Source: Front Public Health. 2026 Jul 2;14:1850081. doi: 10.3389/fpubh.2026.1850081 (PMC13373039; doi:10.3389/fpubh.2026.1850081)
Supplement: Supplementary file 3 [file Table_3.docx]

**Supplementary Table 3.** Between-group comparisons of standardized regression coefficients using the Paternoster Z-test.

| **Service domain** | **No disease vs**  **Single disease** | | **No disease vs Multimorbidity** | | **Single disease vs Multimorbidity** | |
| --- | --- | --- | --- | --- | --- | --- |
|  | **Z** | ***p*** | **Z** | ***p*** | **Z** | ***p*** |
| Q2. Session duration adequacy | −.667 | .505 | 1.608 | .108 | 1.872 | .061 |
| Q3. Program structure appropriateness | 4.058 | .000 | 1.242 | .214 | −.900 | .368 |
| Q4. Perceived usefulness of activities | −2.003 | .045 | 0.924 | .355 | 1.899 | .058 |
| Q5. Group size adequacy | −.422 | .673 | −.730 | .465 | −.459 | .646 |
| Q6. Instructor expertise | .336 | .737 | −2.868 | .004 | −2.940 | .003 |
| Q7. Reservation and participation convenience | −1.295 | .195 | −.416 | .677 | .308 | .758 |
| Q8. Information sufficiency | −.291 | .771 | 2.229 | .026 | 2.260 | .024 |
| Q9. Equipment appropriateness | 2.012 | .044 | −1.455 | .146 | −2.451 | .014 |
| Q10. Environmental comfort and harmony | .167 | .868 | 1.862 | .063 | 1.658 | .097 |
| Q11. Amenities accessibility | -2.086 | .037 | -.806 | .420 | .336 | .737 |

Z = (β₁ − β₂) / √(SE(β)₁² + SE(β)₂²). Standard errors of standardized coefficients were derived as SE(β) = SE(B) × SD(X)/SD(Y), where SE(B) are the standard errors of unstandardized coefficients reported in Tables 5–7, and SD(X) and SD(Y) are the standard deviations of the predictor and outcome variables, respectively. Positive Z indicates the first group's β exceeds the second group's β. No disease: n = 2,455; Single disease: n = 1,378; Multimorbidity: n = 392.
