# Supplementary material for: Predictors of user satisfaction with forest healing services differ by health status
Source: Front Public Health. 2026 Jul 2;14:1850081. doi: 10.3389/fpubh.2026.1850081 (PMC13373039; doi:10.3389/fpubh.2026.1850081)
Supplement: Supplementary file 4 [file Table_4.docx]

**Supplementary Table 4.** Representative quotes by axial category and health status group (translated from Korean).

| **Axial category** | **No disease group** |  | **Chronic disease group** |
| --- | --- | --- | --- |
| 1. **Program time and  operational adequacy** | *I wish the program were a bit longer.* |  | *I would like more frequent sessions.* |
| 1. **Accessibility and  mobility convenience** | *I wish parking facilities were improved so more people could use the program.* |  | *A shuttle bus to the healing center would improve accessibility.* |
| 1. **Basic convenience  infrastructure** | *The restroom is difficult to access.* |  | *It would be better if water were provided; the restroom was inconveniently far away.* |
| 1. **Program content  diversity and depth** | *I wish more emotional and mental content were included in the program.* |  | *I would like to have meditation sessions near a stream where we can hear the sound of water.* |
| 1. **Spatial safety and  environmental improvement** | *I hope the clay path is repaired and extended so we can walk more.* |  | *Slopes or ramps are needed in addition to stairs.* |
| 1. **Information, guidance,  and reservation accessibility** | *It would be helpful to have guidance on what personal items to bring, such as blankets or mats.* |  | *I wish the application process for the forest recreation facility were easier.* |
| 1. **Promotion and  service outreach** | *I hope better promotion will help more people learn about and participate in the program.* |  | *I hope this program becomes more widely known to the public.* |
| 1. **Instructor competence and operational expertise** | *Clearer speech and better use of time for equipment activities would improve the experience.* |  | *The instructor's explanations were too long.* |

All quotes are translated from Korean by the research team. Quotes were selected to represent the most frequently expressed concerns within each axial category. No disease: n = 168 meaning units; Chronic disease (single disease: n = 131; multimorbidity: n = 33; combined: n = 164).
